# Supplementary material for: Preferred Place of End-of-Life Care Based on Clinical Scenario: A Cross-Sectional Study of a General Japanese Population
Source: Healthcare (Basel). 2023 Jan 31;11(3):406. doi: 10.3390/healthcare11030406 (PMC9914905; doi:10.3390/healthcare11030406)
Supplement: Supplementary file 1 [file healthcare-11-00406-s001.zip › healthcare-2159133-supplementary.pdf]

Table S1. The results of the chi-square test and Pearson's residual.

	Home	Nursing home	Medical facility	Total
Cancer	441	97	351	889
	49.61	10.91	39.48	100.00
	282.333	250.000	356.667	
	9.443	−9.677	−0.300	
End-stage heart disease	271	168	450	889
	30.48	18.90	50.62	100.00
	282.333	250.000	356.667	
	−0.674	−5.186	4.942	
Dementia	135	485	269	889
	15.19	54.56	30.26	100.00
	282.333	250.000	356.667	
	−8.768	14.863	−4.642	
Total	847	750	1070	2667
	31.76	28.12	40.12	100.00

The numbers in one cell in rows are N, percentage, expected number, and Pearson's residual.
